# Permeation of Nicotinamide Mononucleotide (NMN) in an Artificial Membrane as a Cosmetic Skin Permeability Test Model

**DOI:** 10.1111/jocd.70222

**Published:** 2025-05-03

**Authors:** Risako Betsuno, Takuya Yamane, Hiroko Tsuji, Yu Nakajima, Momoko Imai, Takeshi Bamba, Susumu Uchiyama

**Affiliations:** ^1^ Department of Biotechnology Graduate School of Engineering, Osaka University Osaka Japan; ^2^ BYU‐Analytica Inc. Osaka Japan; ^3^ Division of Metabolomics/Mass Spectrometry Center Medical Research Center for High Depth Omics, Medical Institute of Bioregulation, Kyushu University Fukuoka Japan

**Keywords:** bioengineering, biological extracts, niacin derivatives, niacinamide

## Abstract

**Background:**

Skin aging progresses owing to intrinsic factors, including chronological alterations, and extrinsic factors, including ultraviolet radiation (UV), smoking, and air pollution. One of the mechanisms of aging is a decrease in NAD^+^ levels in the body, as an increase in NAD^+^ levels prevents skin aging phenotypes. Nicotinamide mononucleotide (NMN) is a precursor of NAD^+^ in the salvage pathway and the primary source of NAD^+^ in mammals.

**Aim:**

This Study Aimed to Evaluate the Permeability, Functionality, and Stability of NMN in Cosmetics.

**Methods:**

The permeation of NMN contained in a yeast‐fermented filtrate (YFF) was evaluated in vitro using artificial skin membranes (Strat‐M). Localization of NMN in the membrane was detected by MALDI‐imaging mass spectrometry (MALDI‐IMS) analysis. Collagen production in dermal fibroblasts was determined using Collagen Type I pro (ELISA). The analysis of the NMN degradation reaction and the determination of the half‐life of NMN in the YFF were performed using high‐performance liquid chromatography.

**Results:**

NMN Permeated and Was Detected Only in the Papillary Dermis Region of the Membrane by MALDI‐IMS. Furthermore, Collagen Type 1 Production Was Increased in Human Fibroblasts When Treated With NMN. On the Other Hand, the NMN Degradation Reaction Was a First‐Order Reaction, and the Half‐Life of NMN in the YFF Was Approximately 7 months at 20°C. These Results Suggest That NMN Was Relatively Stable in the YFF and Did Not Reach the Subcutaneous Tissue, and That NMN Increased Collagen Production by Fibroblasts in the Papillary Dermis.

**Conclusions:**

NMN in the YFF permeated artificial skin membranes, and NMN contained in YFF enhanced collagen Type 1 production in fibroblasts. Therefore, the YFF, containing NMN, may be a potential cosmetic ingredient.

## 1 Introduction

The skin is the largest organ in the human body. Specific functions of the skin include thermoregulation, immunological surveillance, and protection from external chemical, mechanical, and pathogenic stressors. The skin also regulates the permeation of various compounds as a barrier. Therefore, it is important to evaluate skin permeation in vitro during cosmetic development to ensure safety and functionality. However, human skin is difficult to obtain, and animal experiments have raised ethical issues in the cosmetic industry. Accordingly, the development of artificial membranes and 3D cultured human skin models as substitutes for human skin has progressed. Artificial membranes have attracted attention, especially because they have the advantages of easy preparation, less storage space, and comparatively low cost [1]. The artificial membrane is designed to mimic human skin and provide an alternative method to evaluate drug permeability through the skin [2, 3, 4].

NAD^+^ is involved in various biological processes, such as energy metabolism [5]. NAD^+^ levels decrease with age, leading to the hypothesis that decreased NAD^+^ levels cause age‐associated functional decline and disease [5, 6]. Therefore, it is important to restore NAD^+^ levels.

In the skin, it has also been shown to decrease inflammation by restoring the decrease in NAD^+^ [7]. However, direct absorption of NAD^+^ is impossible, due to a cellular process involving intake of NAD^+^ intermediates followed by intracellular synthesis of NAD^+^ from the intermediates [6]. Therefore, the intermediates that can synthesize NAD^+^ have been focused on.

NMN is one of the precursors of NAD^+^ through the salvage pathway, which is the primary source of NAD^+^ in mammals [5]. In previous research, it was found that NMN was effective for the skin, in terms of enabling melanin production to reduce through downregulated genes that are mainly involved in melanogenesis‐related processes [8], and blocking skin damage induced by UVB exposure in mouse skin [9]. These studies were performed by oral gavage or intraperitoneal injection, and NMN transdermal absorption needs to be better studied.

Recently, the cosmetics industry has been using culture filtrates of microorganisms and cells. Yeast‐fermented filtrate (YFF) contains small amounts of yeast‐derived substances. These components are amino acids, carbohydrates, proteins, and organic acids, which may improve the stability of NMN. Previous studies have reported that YFF enhances skin barrier function by increasing the expression of protective proteins in cells and inhibiting inflammation, epidermal damage, and melanin production by yeast substances [10, 11, 12]. However, few studies have examined the effects of NMN and the YFF on the skin.

In this study, Strat‐M, a skin‐mimicking membrane, was used for testing permeability and localization of NMN. The permeability of NMN contained in the YFF was measured using reversed‐phase high‐performance chromatography (RP‐HPLC). Furthermore, NMN localization in the skin layer of the Strat‐M membrane was analyzed using MS imaging spectrometers. The stability of NMN in the YFF was also measured using RP‐HPLC, and the NMN degradation products were identified. In this experiment, we used yeast fermentation filtrate containing NMN rather than NMN alone to improve NMN's stability and synergize its efficacy.

## 2 Materials and Methods

### 2.1 Materials

Nicotinamide mononucleotide and nicotinamide were purchased from Oriental Yeast Co. Ltd. (Shiga, Japan) and Nacalai Tesque Inc. (Kyoto, Japan). Yeast‐fermented filtrate (YFF) was obtained from Besbio Co. Ltd. (Mie, Japan). 2,5‐Dihydroxybenzoic acid (DHB) and Polyethylene Glycol (PEG; average M_n_ 300) were purchased from Merck (Darmstadt, Germany). Indium tin oxide (ITO)‐coated glass slides (100 Ω/sq. without adhesive material coating) for MALDI‐MSI were purchased from Matsunami Glass (Osaka, Japan). The 4% carboxymethyl cellulose embedded media was purchased from Section‐Lab (Hiroshima, Japan). 0.1% formic acid (KANTO CHEMICAL Co. Inc., Japan), nicotinamide (NAM; from NACALAI TESQUE Inc., Japan). Procollagen Type I C‐peptide (PIP) EIA Kit was purchased from Takara Bio Inc. (Shiga, Japan).

### 2.2 In Vitro Permeation Studies of NMN Contained in YFF

The in vitro permeation of NMN contained in the YFF through a skin‐mimetic model was evaluated using a Franz diffusion cell system (Perme Gear Inc., USA) with a Strat‐M membrane (Merck, Germany). In the Franz diffusion cell system, the receptor compartment was filled with D‐PBS, and 1 mL of the NMN formulation (10 mg/mL in YFF) was charged into the donor compartment. The receptor solution was maintained at 37°C while agitating using a stirrer bar and magnetic stirrer throughout the experiment. After 24 h, 300 μL of the receptor solution and the entirety of the donor solution were collected. Further, the membrane was washed with distilled water and subjected to extraction with 5 mL of 0.1% formic acid in distilled water for 24 h. Thus, extracted samples were subjected to RP‐HPLC high‐performance liquid chromatography for NMN quantification.

### 2.3 Stability Test of NMN in YFF

To examine the stability of NMN in the YFF, a mixture containing 10 mg/mL NMN dissolved in the YFF was prepared. Three aliquots of the mixture were incubated at 40°C, 60°C, and 80°C, respectively, and sampled after 2, 4, 6, 24, 48, and 72 h. The samples were diluted 1000 times with 0.1% formic acid in distilled water and filtrated through a 0.2 μm membrane. The amounts of NMN and NAM in the samples were quantified by UHPLC. The UHPLC consisted of a Thermo Scientific Vanquish UHPLC system equipped with a binary pump, an autosampler permanently cooled to 4°C, a Supelco Discovery HS F5‐3 column (dimensions: 150 × 4.6 mm, particle size: 3 μm; Merck, Germany), and a diode array detector. Mobile phases A and B were 0.1% formic acid in distilled water and 0.1% formic acid in acetonitrile, respectively. The UHPLC was run at a flow rate of 0.25 mL/min with the gradient involving: 0–5 min, 0% buffer B; 5–15 min, 40% buffer B; 15–18 min, 60% buffer B; and 18–25 min, 100% buffer B, with an injection volume of 2 μL at a wavelength of 265 nm and at an oven temperature of 40°C. Peaks were quantified based on the peaks at 2 and 4 min by comparing the peak areas with pure standards at known concentrations.

### 2.4 Membrane Sectioning and Mounting

Before sectioning, membrane samples were embedded in 4% CMC in cryomolds (Sakura Finetek Japan, Tokyo, Japan). The samples were put into a deep freezer (−80°C) until complete freezing. Frozen serial 15 μm sections were obtained by cutting the embedded sample on cryofilm at −22°C with a Microtome (CM1950; Leica, Nussloch, Germany) and mounted on ITO‐coated glass slides with conductivity tape for MALDI‐MSI. The sections were dehydrated in a 50 mL conical tube containing silica gel and stored until the analysis at room temperature.

### 2.5 MALDI‐MSI Analysis

MALDI‐MSI experiments were performed on a MALDI trapped ion mobility time‐of‐flight mass spectrometer (timsTOF fleX; Bruker Daltonik Bremen, Germany) equipped with a 10 kHz Nd: YAG laser (*λ* = 355 nm). The pixel size was 10 μm in single laser beam scanning mode, and each pixel was irradiated 250 times at a repetition rate of 10 kHz. The red phosphorus cluster peak was used for the m/z calibration. Mass spectra were acquired in the TIMS positive ion mode. The ion mobility range was 0.1 to 1.2 (1/K0) and the ramping time was 300 ms. The spatial resolution of imaging measurements was performed at 10 μm. After the measurement, the data were analyzed using SCiLS Lab2023a (Bruker Daltonics, Bremen, Germany) for visualization. The m/z value of the target was 335.06384, which was derived from [M + H]^+^. The mobility value 1/k0, previously obtained using the standard, was 0.792 (CCS: 165.1 [Å2]). Acquired data visualization used SCiLS Lab2023a to reconstruct ion images based on data extracted from target m/z ± 2 m Da and 1/k0 ± 0.01 range.

### 2.6 LC–MS/MS Analysis

Samples were placed in a Discovery HS F5‐3 column (2.1 × 150 mm) containing mobile phase A (0.1% formic acid) and mobile phase B (0.1% formic acid in acetonitrile) and analyzed using A dual UHPLC pump (Vanquish, Thermo Fisher Scientific, CA, USA) coupled with an orbitrap mass spectrometer (Q‐Exactive, Thermo Fisher Scientific, CA, USA). The column was run at a flow rate of 0.25 mL/min, according to the following time program: 0–5 min, 0% B; 5–15 min, 0%–40% B; 15–15.1 min, 40%–100% B; 15.1–18 min, 100% B; 18.1–25 min, 0% B. The column temperature was maintained at 40°C. The eluate was subjected to electrospray ionization (the spray voltage 4 kV, the capillary temperature 320°C). The mass spectrum of the eluate was recorded between m/z 150 and 1000 in positive ion mode. Data analysis was performed using Compound Discoverer 3.1 software (Thermo Fisher Scientific).

### 2.7 Cell Culture

Normal human dermal fibroblasts (NHDF) were cultured in a fibroblast basal medium mixed with SupplementMix (PromoCell, Germany). These cells were seeded in 24 well plates and incubated for 1 day. After that, 5 μL of NMN in the YFF was added to each well, such that the final concentration of NMN in the medium was 1 mM. After 1 day of incubation, the culture supernatant was collected and samples were stored at −80°C.

### 2.8 Collagen Type I Production Using ELISA

ELISA was performed according to the manufacturer's instructions. Briefly, in a 96‐well plate, 20 μL/well of each sample and 100 μL/well of standard antibody solutions were added, and the reaction was carried out for 3 h at 37°C. The reaction solution was discarded, the plate was washed 4 four times with PBS, 100 μL/well of a substrate solution was added, and the reaction was carried out for 15 min at room temperature. The stop solution was then added to the plate and mixed thoroughly. Absorbance was measured at 450 nm.

### 2.9 Statistical Analysis

Quantitative data are expressed as mean ± S.E. Statistical analyses were performed using Statcel4 software (OMS, Tokyo, Japan). Differences between the two groups were evaluated using analysis of variance (one‐way ANOVA) followed by an unpaired Student's *t*‐test.

## 3 Results

### 3.1 Permeation of NMN Contained in YFF

**FIGURE 1:**
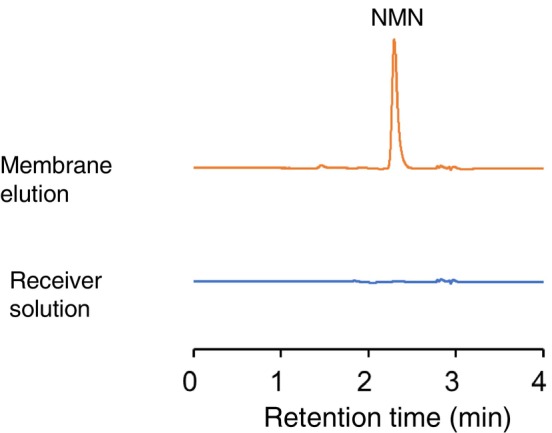
Chromatograms of membrane elution (A) and receptor compartment solution (B).

To evaluate the permeation of NMN contained in the YFF, the eluted fraction from the membrane and the fraction from the receptor compartment were analyzed using UHPLC. As shown in Figure 1, NMN was detected in the eluted fraction from the membrane but not in the fraction from the receptor compartment.

### 3.2 Localization of NMN in the Membrane Using MALDI‐MSI Analysis

**FIGURE 2:**
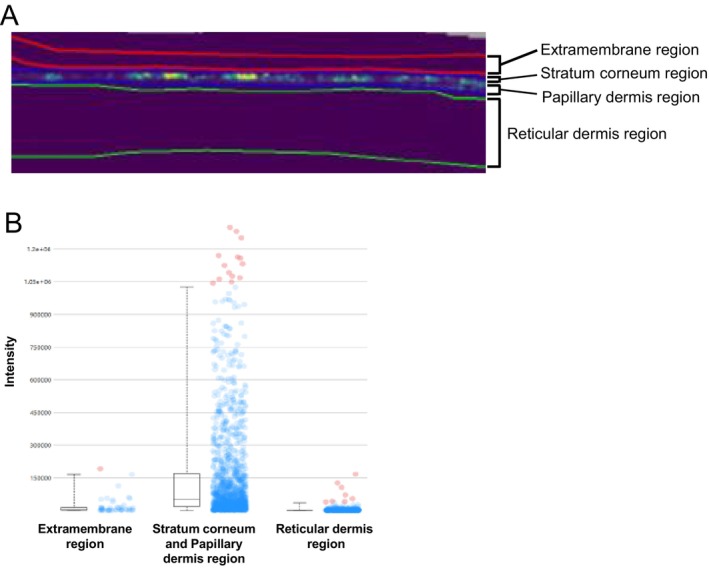
Localization analysis of NMN in membranes using imaging mass spectrometry. Mass imaging of NMN in the membrane (A) and quantified data (B) using timsTOF fleX.

Because NMN was detected in the fraction eluted from the membrane, the localization of NMN in the membrane was observed. MALDI‐MSI analysis revealed that NMN was localized in the stratum corneum and papillary dermis regions but not in the reticular dermis region (Figure 2).

### 3.3 Effect of NMN on Collagen Synthesis

**FIGURE 3:**
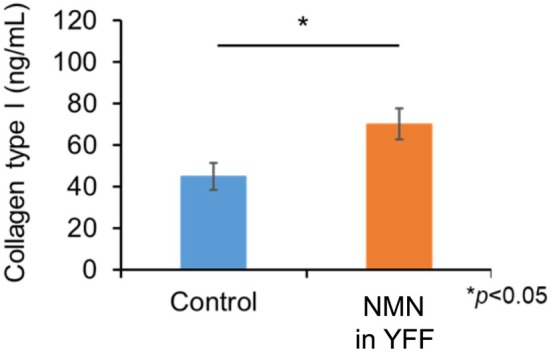
Enhanced production of collagen type I in fibroblast cells treated with NMN in YFF *n* = 5, *p < 0.05. Cells in the control group were treated with YFF that did not contain NMN.

According to the NMN permeation results, NMN could permeate the papillary dermis region of the artificial membrane. Fibroblasts are present in the papillary dermis and produce collagen that is able to prevent wrinkling and sagging. Therefore, we investigated the effect of NMN on collagen production in fibroblasts. Fibroblasts were exposed to NMN, and collagen type I production was analyzed using ELISA. As shown in Figure 3, collagen type I production in fibroblasts treated with NMN was significantly higher than that in control fibroblasts. Therefore, NMN enhanced collagen type I production in fibroblasts.

### 3.4 Stability of NMN in YFF

**FIGURE 4:**
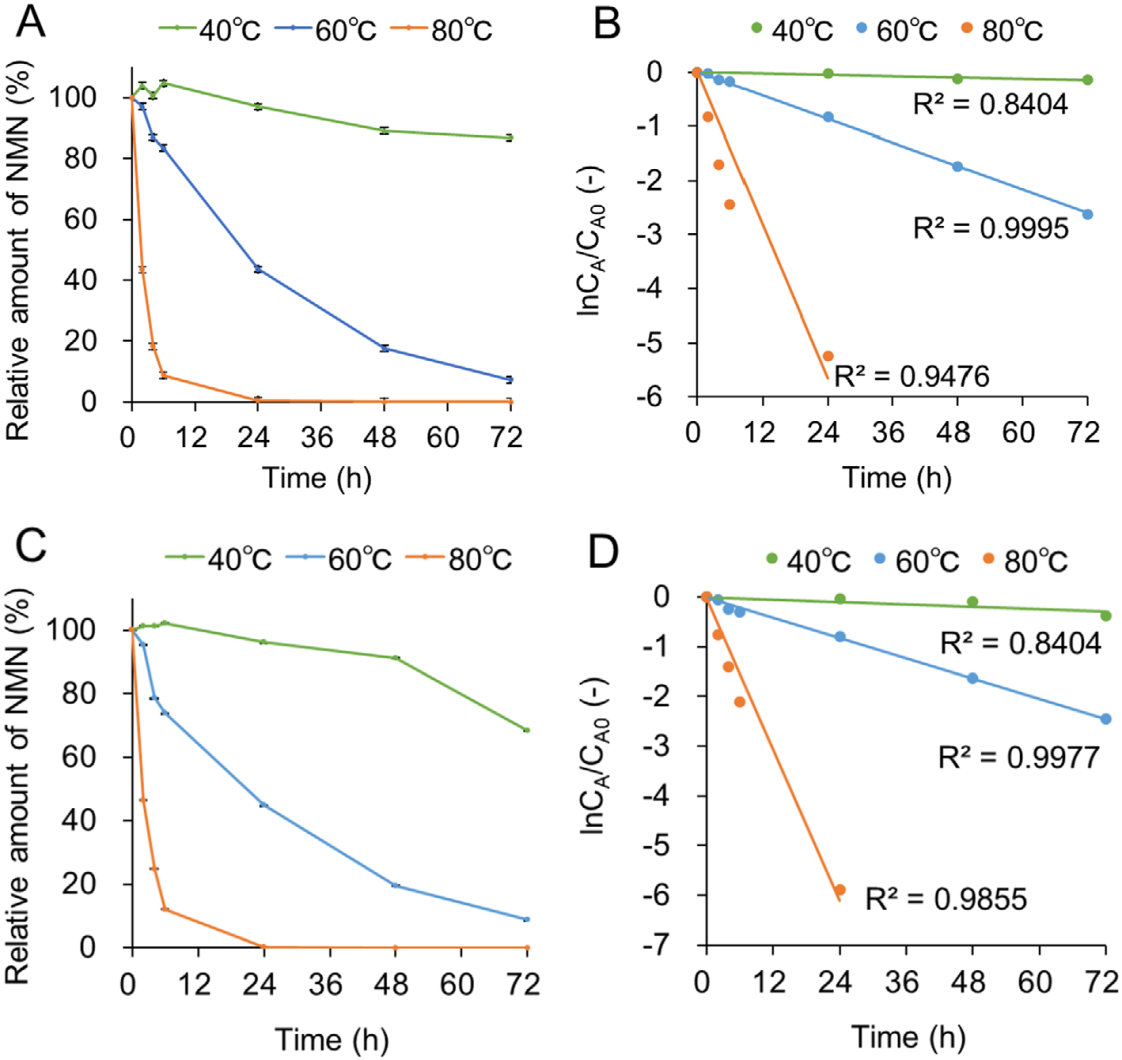
Stability of NMN in YFF. NMN amount at 0 h was defined as 100%, and the relative amounts of NMN over time in YFF (A) and water (C) are shown. The first‐order reaction rate constant fitting is shown in (B) and (D).

The stability of NMN in the YFF was measured at high temperatures for 72 h. As shown in Figure 4A, 90% of NMN was stable at 40°C for 72 h. On the other hand, 90% of NMN was degraded at 60°C for 72 h. In addition, NMN was completely degraded at 80°C for 48 h. First‐order reaction rate constant fitting revealed that NMN degradation in the YFF was a first‐order reaction (Figure 4B). NMN stability in water was measured under the same conditions as in the YFF. As shown in Figure 4C, 75% of NMN was stable at 40°C for 72 h. On the other hand, 90% of NMN was degraded at 60°C for 72 h. In addition, NMN was completely degraded at 80°C for 48 h. First‐order reaction rate constant fitting revealed that NMN degradation in water was also a first‐order reaction (Figure 4D). The activation energy was calculated using a first‐order reaction fitting line. The activation energies for each NMN degradation reaction in the YFF and water were 117 and 114 kJ/mol, respectively.

### 3.5 Identification of NMN Degradation Products Using LC–MS/MS

**FIGURE 5:**
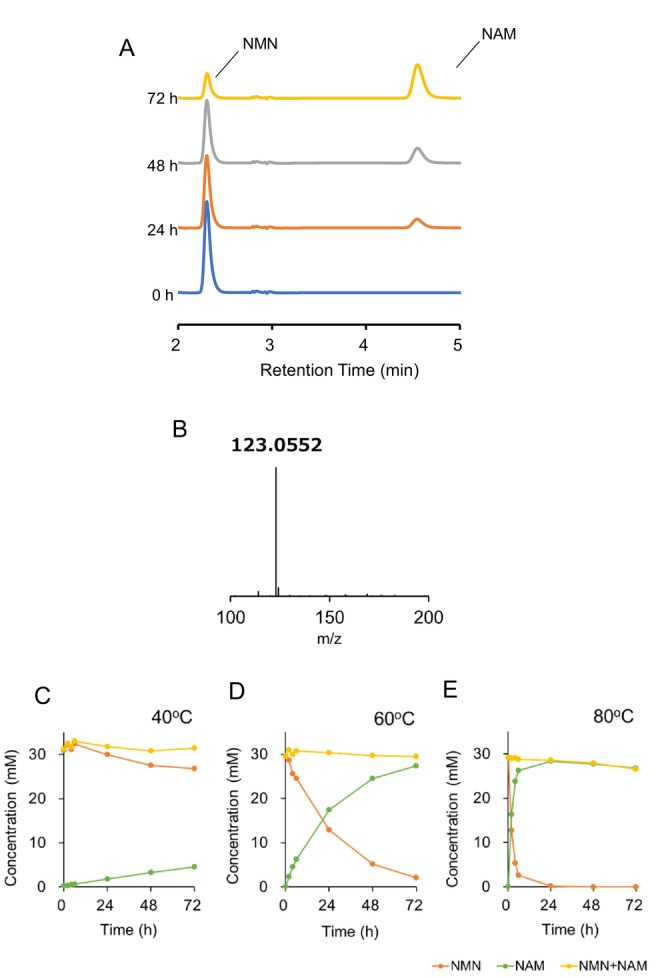
Changes in NMN and NAM concentrations in YFF at various temperatures. (A) Chromatograms of NMN and NAM in the YFF incubated at 40°C for 72 h using RP‐HPLC detected at 265 nm. (B) MS/MS spectrum of NAM peak. NMN and NAM concentrations at incubation temperatures of 40°C (C), 60°C (D) and 80°C (E).

Measurement with high‐performance liquid chromatography was conducted after incubation of NMN in the YFF at 40°C for 72 h. As shown in Figure 5A, product peaks were detected after incubation with NMN in the YFF, and the concentration of NMN decreased, whereas the concentration of the product increased in a time‐dependent manner. LC–MS/MS analysis revealed that the product was nicotinamide (NAM) (Figure 5B). The production of NAM at 40°C, 60°C, and 80°C was faster as the reaction temperature increased (Figure 5C–E).

## 4 Discussion

In this study, skin permeation of NMN contained in YFF was performed using an artificial membrane as a skin model. NMN in the YFF permeated the membrane and localized to the papillary dermis region of the membrane. Furthermore, NMN in the YFF permeated into the upper dermal region of the membrane. These results indicate that NMN can act on fibroblasts in the upper dermis. It is important to evaluate the permeation of compounds into the skin to ensure the safety and effectiveness of cosmetics. Thus, in vitro permeability evaluation is necessary because animal experiments for cosmetic development are prohibited by the European Cosmetic Regulations. In vitro permeability evaluation is commonly performed on excised human or animal skin, three‐dimensional cultured cell skin models, or skin‐mimicking artificial membranes [13]. In the present study, it was found that small molecular weight compounds such as NMN in YFF can be evaluated for permeability using artificial membranes, and their safety can be confirmed.

There are three pathways through which compounds permeate the skin: intercellular, transcellular, and transappendageal. The transappendageal pathway is not considered an important compound permeation pathway because sweat glands and hair follicles occupy only 0.1% of the surface area of the skin [14]. Although the transcellular pathway is also possible, the intercellular pathway is more dominant for low‐molecule compounds.

The permeation process consists of the release of compounds from the formulation, diffusion into the epidermal stratum corneum, and diffusion/permeation into deeper layers and tissues. This process depends on the solubility and diffusivity of the permeation compounds. The parameters used to predict the solubility and diffusivity include the partition coefficient, molecular size, and solubility in water. The first permeation step into the skin is the partitioning of compounds into the epidermal stratum corneum lipid domain; in this step, the partition coefficient contributes significantly. In particular, skin permeability is enhanced as the compound becomes more lipophilic, suggesting that the optimal logP(o/w) is 2–3 [15]. Molecular size and solubility, which are the other main parameters, are important factors. It has been shown that there is an inverse correlation between molecular size and skin permeability, and the molecular size of the compounds used for transdermal adsorption is often less than 500 Da [15]. Hence, NMN can permeate the skin because its size is approximately 334 Da. In addition, the lower the solubility of a molecule in water, the higher its solubility and diffusion in the intercellular domain. Due to being highly hydrophilic and not capable of diffusing easily, NMN would have remained in the upper dermal region of the membrane.

On the other hand, the stability of NMN in YFF under high‐temperature conditions showed that the NMN degradation reaction was a first‐order reaction and the half‐life of NMN in YFF was approximately 7 months at 20°C. In addition, nicotinamide is an NMN degradation product. The NMN degradation reaction in water was also a first‐order reaction and the activation energies were the same for water and YFF. These results indicate that the NMN degradation reaction was mainly caused by the attack of water molecules. To keep NMN stable in YFF, it is necessary to keep the storage temperature low, adjust the pH, and encapsulate it into a liposome.

Furthermore, collagen Type 1 production was increased in human fibroblasts treated with NMN. These results suggest that NMN increases collagen production by fibroblasts in the papillary dermis. YFF contains amino acids, carbohydrates, proteins, and organic acids. These YFF‐containing compounds may affect collagen synthesis and NMN permeability. Further research is needed to clarify this phenomenon.

Finally, nicotinamide, a breakdown product of NMN, has been reported to promote skin whitening by inhibiting the transfer of melanosomes from melanocytes to keratinocytes in the body [16]. In clinical trials, the application of nicotinamide significantly reduced the area of detected facial blemishes and the appearance of pigmentation [17]. Furthermore, some clinical trials have reported that nicotinamide intake significantly improves the appearance of the skin [18]. These previous studies suggest that nicotinamide, a breakdown product of NMN, may also be applied as a compound that reduces the signs of skin aging, along with NMN.

## 5 Conclusions

In conclusion, NMN in the YFF can permeate the stratum corneum and papillary dermis. Furthermore, collagen type I production in fibroblasts was enhanced by NMN in the YFF. Therefore, NMN in the YFF is useful as a cosmetic product for maintaining skin quality.

## Author Contributions

T.Y., T.B., and S.U. conceived and designed the study. R.B., H.T., Y.N., and M.I. conducted the experiments. R.B. and T.Y. analyzed the data. R.B. and T.Y. prepared the manuscript. All the authors have read and approved the final manuscript.

## Conflicts of Interest

The authors declare no conflicts of interest.

## Data Availability

Research data are not shared.
